# Smart Video Surveillance System Based on Edge Computing

**DOI:** 10.3390/s21092958

**Published:** 2021-04-23

**Authors:** Antonio Carlos Cob-Parro, Cristina Losada-Gutiérrez, Marta Marrón-Romera, Alfredo Gardel-Vicente, Ignacio Bravo-Muñoz

**Affiliations:** Department of Electronics, University of Alcalá, Alcalá de Henares, 28801 Madrid, Spain; antonio.cob@edu.uah.es (A.C.C.-P.); cristina.losada@uah.es (C.L.-G.); marta.marron@uah.es (M.M.-R.); ignacio.bravo@uah.es (I.B.-M.)

**Keywords:** machine learning, embedded systems, video-surveillance, mobilenet-SSD, vision processor unit, edge node, artificial intelligence

## Abstract

New processing methods based on artificial intelligence (AI) and deep learning are replacing traditional computer vision algorithms. The more advanced systems can process huge amounts of data in large computing facilities. In contrast, this paper presents a smart video surveillance system executing AI algorithms in low power consumption embedded devices. The computer vision algorithm, typical for surveillance applications, aims to detect, count and track people’s movements in the area. This application requires a distributed smart camera system. The proposed AI application allows detecting people in the surveillance area using a MobileNet-SSD architecture. In addition, using a robust Kalman filter bank, the algorithm can keep track of people in the video also providing people counting information. The detection results are excellent considering the constraints imposed on the process. The selected architecture for the edge node is based on a UpSquared2 device that includes a vision processor unit (VPU) capable of accelerating the AI CNN inference. The results section provides information about the image processing time when multiple video cameras are connected to the same edge node, people detection precision and recall curves, and the energy consumption of the system. The discussion of results shows the usefulness of deploying this smart camera node throughout a distributed surveillance system.

## 1. Introduction

Nowadays, deep learning has shown great advantages in several research fields, for example finance [1], medicine [2], automatic modulation classification in cognitive radios [3], and many others. In particular, computer vision was the first research field in which deep learning took place [4]. New processing methods based on deep learning are replacing traditional computer vision algorithms relying on physical representations, models, functions with some level of meaning deep learning vs. traditional computer vision [5]. The more advanced systems are able to process huge amounts of data in large computing facilities. Current challenges are related to training the machine learning system with enough information which requires the labelling of those data. On the other hand, in this paper, we focused our attention on smart camera nodes distributed along a surveillance area which are far from that approach.

The modernisation of technologies at both the software and hardware level has allowed the design of smart video systems capable not only of managing the video feeds from a closed camera circuit but also analysing and extracting information in real time from the video streams.

These embedded systems are installed in both public and private locations, being able to control the number of people in an area, crowds movement, detecting anomalous behaviours, etc. Most of these systems are centralised, executing the computer vision algorithms in one single location. This central processing system receives and processes the information from all the camera network. Old systems did not process video information and required the operator’s analysis, who was duty bound to carefully monitor any camera and whose analysis efficiency may decrease due to fatigue and boredom [6,7].

The modern video processing centralised systems [8,9] store and process the video information retrieved from the camera network. Only some alerts or video-clips are shown to the central console/operator without requiring a high level of attention into the management of the surveillance system.

On the other hand, the emergence of the Internet of Things (IoT) and the computing on the edge nodes [10], has led to the appearance of many research works that propose distributed video-surveillance systems based on this concept [11]. Hence, the intelligence of the system is distributed in multiple nodes, where each one can include a camera and a processing system that performs simple tasks before sending the information to the operator, facilitating its work.

For more complex tasks, current detection algorithms for computer vision include deep neural networks (DNNs). To execute DNNs, a high-end hardware system with high computing power, such as graphical process units (GPUs), is usually required. Apart from being expensive, these hardware modules have a high energy consumption, and it can be difficult to embed them in the distributed smart camera nodes.

In this context, our paper presents a smart video-surveillance system for detecting, counting and tracking people in real time, in a embedded hardware system using new vision processing units (VPUs) hardware modules. The paper describes both the hardware architecture used in the embedded system and the developed computer vision algorithm for detecting, tracking and counting people in real time. This system can be easily installed and configured to work as a smart camera edge node in a distributed video-surveillance system.

The proposed system is based on a low-cost embedded platform UpSquared2 [12], that includes a VPU, the Myriad-X [13]. This allows the parallelisation of the developed algorithms to allow them to work in real time, with a reduced power consumption.

A MobileNet-SSD architecture [14] has been selected for the task of tracking and detecting people. Furthermore, a bank of Kalman filters allows people tracking and counting. We evaluated the performance of the system, making a comparison with other algorithms and extracting the values of the edge node related to performance, computer power and consumption, making a pipelined architecture capable of processing up to 12 video streams simultaneously.

The rest of the paper is organised as follows. Section 2 presents a study of the main previous works related to people detection and tracking, as well as different edge computing hardware and software tools. Then, Section 3 and Section 4 describe the hardware architecture and the developed algorithms. After that, Section 5 explains the main experimental results, and finally conclusions and future work are presented in Section 6.

## 2. Previous Related Works

### 2.1. Performing AI on Embedded Devices

The main contribution of the paper is to build a smart camera system capable of processing videos locally, at the edge nodes, using embedded devices. For this reason, we need to review the design and capabilities of constrained devices to analyse if they can execute real-time artificial intelligence processing. Shi [15] defines edge computing as “*enabling technologies that allow computing to be performed at the edge of the network, on downstream data on behalf of cloud services and on upstream data on behalf of IoT services*”. This allows using the full potential offered by artificial intelligence, without losing computing capacity in the processor.

There is a variety of devices on the market that allows bringing artificial intelligence to the edge. The best known acceleration coprocessor is the GPU. Most GPUs are designed for being used as part of a desktop computer, but in recent years, different embedded development kits have emerged, including a GPU, such as the Jetson AGX Xavier [16], or the Jetson Nano [17], mainly oriented towards autonomous systems. Another type of technology for hardware acceleration is the Coral [18] family provided by Google. It is a set of devices that can be connected via USB and generate an acceleration system for tensor-oriented architectures.

The latest technology for the development of video-surveillance systems is the VPU, developed by Intel, and focused on the parallel processing of neural networks. VPUs are oriented towards high-speed inference processing with very low power consumption, being able to implement this type of device in embedded systems, drones, or systems powered by external power supplies. Intel has already released three versions of the device, with the Myriad-1 and Myriad-2 being discontinued and now only Myriad-X being on the market. The VPU can be connected via USB (NCS2) or PCIe.

This type of technology is becoming increasingly valuable in the development of smart systems, as evidenced by Kristianin et al. in [19] by designing an object classification system, or Adnan et al. in [20] with the optimisation of a detection system on a Rasberry. Moreover, in the field of astronomy, Surabhi Agarwal et al. [21] describe the design of a star tracker using an NCS2.

### 2.2. People Detection

Object detection is defined as the ability to recognise and locate a particular type of “object” in an image. In this context, the literature related to people detection in video-surveillance applications is very extensive [22,23]. Among the different proposed methods, two large groups can be distinguished: those that are based on classical methods, which make use of mathematical techniques and people feature modelisation, and modern approaches based on deep learning.

Belonging to the image pattern recognition classical methods, one of the most popular is the one proposed by Dalal and Triggs [24]. This algorithm obtains image features using a histogram of gradient (HOG) and then applies a support vector machine (SVM) classifier (widely used in other applications such as natural language [25] or speech recognition [26]). The authors in [27] also proposed the use of HOG features for people’s head and shoulders detection, combined this time with a classifier based on principal components analysis (PCA).

There are other video-surveillance proposals that address both people detection and tracking using different techniques. In this context, the proposal in [28] presents a method that combines a Kalman filter and HOG features, whereas [29] describes an approach based on image segmentation and a Kalman filter.

In recent years, the use of RGBD cameras [30] that provide both RGB images and depth information (distance from each 3D point to the camera) using different technologies, such as stereo vision, or time of flight, has impressively increased. Due to this, numerous works have appeared that use this information (instead of only RGB images) for people detection. Some of these works again use HOG features obtained from RGBD data [31,32], whereas other approaches are based on PCA [33]. Furthermore, there are also works that use only depth information to detect people in a robust way while preserving their privacy [34,35].

The improvements in the processing systems’ capacity has led to a change of paradigm in computer vision. Particularly, image classification and object recognition have embraced the use of neural networks. One of the first networks used was the so-called AlexNet in 2012 [36]. In addition, this type of network can be used for the detection of specific elements, such as pedestrians, as shown in the paper [37], which uses a network based on the detection of pedestrians, which consists of extensive part detectors.

This type of network uses sets of convolutional layers that extract the data of an image, which are used by a dense neural network to classify the image. To train these networks, datasets labelled [38,39,40] are used, allowing to obtain classifiers with a high capacity to generalise and precision values far beyond those given by classical methods. These datasets must be of large size for correct training, and in the case that it is not large enough, there are methods such as [41], which allow to improve the training with a smaller number of images reducing the problem of overfitting. In spite of the good results both in general and in precision, the high computational cost must be taken into account when working with this type of network, making it expensive and difficult to work in real time.

To achieve real-time execution and speed up the calculations, several mathematical models have been proposed. These are commonly named one-shot algorithms, due to their ability to locate the region with the more relevant information within the image. Among these algorithms, the most known are R-CNN [42], fast R-CNN [43], faster R-CNN [44], SSD (single shot detection) [45] and YOLO [46]. The last three can process images in real-time in constrained devices. We selected SSD for our people detection algorithm.

These algorithms are combined with CNNs to develop systems with high accuracy and that can run in real time. The resulting architectures, when compared with the classical methods, are slower in training but have better results both in time and in accuracy in the inference. It is possible to find systems as Masuzawa et al. [47] which adopted the YOLO object detection method through a Darknet framework to generate the bounding box of detected people.

Concerning the detection of people by artificial intelligences in embedded systems, there is a great variety of articles related to this topic. This is due to the great variety of existing AIs, the number of embedded systems on the market, as well as the way to implement one in another. In the literature, there are a lot of systems implemented in Rasberry Pi, which is due to its price and its computing capacity. For this device, we find people detection systems using classical AI such as the use of HOG+SVM with thermal images in the case of the paper [48] or by using deep learning models as in the case of [49] which uses it for pedestrian detection in cars. Apart from the rasberri pi, there are other hardware systems such as the NVIDIA Jetson Nano, which contains an internal GPU that allows the inference of networks with ease as in the case of [50].

## 3. Embedded Processing System: Hardware and Software Components

Since one of the requirements of the proposed systems is its portability and flexibility for deployment, the selection of the hardware embedded platform was one of the main tasks addressed in the research work. Below, the hardware components characteristics and the software framework used later are briefly explained.

The embedded platform selected for the smart node is the UpSquared2 system [12], a specialised hardware characterised by: an Intel Atom x7-E3950 microprocessor, an 8 Gigabyte (GB) RAM memory, a 64GB embedded MultiMediaCard (eMMC) ROM. This also includes the deep learning module of Intel Movidius Myriad X VPU [13], a System-On-Chip (SoC) that can be used for accelerating AI inference with a low-power footprint.

The objective of the Myriad-X device is to process DNNs’ inference at high speed and with the lowest possible power consumption. According to the description given by the manufacturer, the architecture of the device allows it to perform more than 4 trillion operations per second (TOPS). This amount of FLOPS is achieved thanks to the combination of the neural compute engine and the 16 128-bit VLIW SHAVE (streaming hybrid architecture vector engine) processors that make up the device. In addition, the system is composed of two LEON4 Cores CPUs (RISC; SPARC v8). As previously mentioned, this device enables focusing on achieving high processing speeds in the inference with low power consumption, resulting as perfect for developing an inference in the limit and in a battery-powered embedded system.

**Figure 1:**
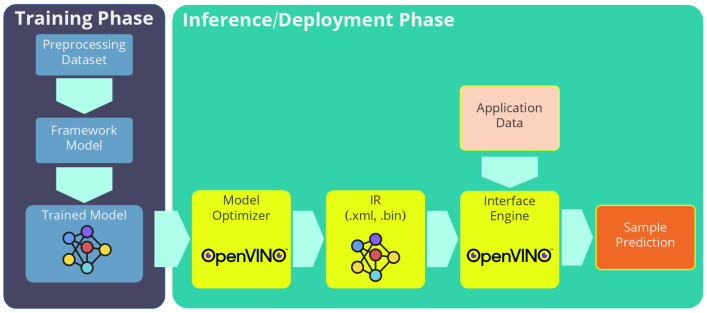
OpenVino structure.

To execute AI algorithms in the VPU, the OpenVino [51,52] framework has been used, which eases the optimising and deployment of CNNs. The OpenVino framework includes two different tools, as shown in Figure 1: the model optimizer and the inference engine. These tools allow optimising the model and executing it in different hardware platforms such as Intel CPUs, VPUs or GPUs, reducing the execution time. One of the advantages of this framework is that it can be installed in any device that meets the minimum requirements. This also allows the installation of these two modules separately, being able to have the model optimizer in the PC used to train the network and the inference engine in the embedded system.

The Model Optimizer is an application that runs on command line and that allows to adjust and optimise the neuronal models to achieve an acceleration in the inference of the system. The OpenVino model optimizer can work with different libraries such as Tensorflow, Pytorch or Caffe. The model optimizer provides the intermediate representation (IR) files. These files are one with an extension .xml which defines the layers, sizes and connections of the architecture and a file .bin, which defines the weights of each parameter of the architecture.

Regarding the inference engine, as mentioned above, this OpenVino module can be installed on any device, independently of the model optimizer. This module loads the IR files and runs the inference on the hardware selected by the plugging. It can be run on CPU, VPU or GPU. In addition, the inference engine is in charge of balancing the inference load so as not to overload any device. Thus, the load is distributed between the CPU cores or between a set of VPUs.

## 4. AI Image Processing to Detect and Track People at the Edge Node

**Figure 2:**
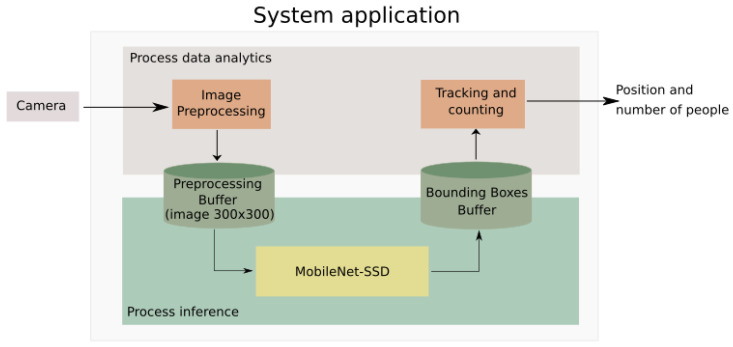
Data flow and multiple thread processing.

One of our research goals was to design an AI application which could detect, track and count people in an embedded system. The proposed architecture (Figure 2) was based on the parallelisation of several processes in order to use the hardware modules available in the most efficient way. The analysis was divided into two processes, which communicate through the use of independent buffers.

In the first process, the so-called data analytics process, the preprocessing of the image and the post-processing of the information returned by the AI inference engine were carried out. Before the preprocessing of the image, different algorithms such as noise reduction, image edge detector [53] or any image enhance low-level pixel processing could be applied. As this low-level preprocessing is dependent on the real scenario, and due to the extra computational cost this preprocessing would add, the preprocessing has been reduced to a minimum: ROI selection, image resize to 300 × 300 pixels and (−1.0, +1.0) range normalization of pixel values, in order to adapt the data to the requirements for the input of the first MobileNet-SSD layer.

The post-processing consists of the reorganisation of the data generated by the MobileNet-SSD network, which contain the bounding boxes of where it has predicted that people are located and then that information is analysed by a Kalman filter bank that predicts movements and future overlaps, returning a more optimal and reliable result. This first process was entirely executed on the CPU of the system. The second process that confers the system is the one that performs the network inference. The inference is designed to be executed at the edge using a VPU. However, there is also the option of running it on a CPU. Once the MobileNet-SSD performs the inference, it returns and stores the bounding boxes in a common buffer.

Figure 2 shows a schematic of the flows that compose the main system. By using elements such as the VPU, more than one inference could be implemented at the same time, being able to process more than one video stream. In the case of using a CPU, the capacity to perform more than one inference is determined by the CPU and the number of cores it contains.

Despite the fact that these processes are pipelined for one single video feed, also several video streams might be considered in the processes pipelining. In the case of the MobileNet-SSD inference, when executed by the VPU, it allows the inference of up to four video streams in an efficient way. This process is explained in more detail in later sections. Depending on the hardware limitations, a higher or lower level of parallelisation can be achieved, which is able to interleave the different processes that make up the system to enable the processing of multiple video streams at the same time.

In the following sections, we discuss the people detection with a MobileNet-SSD and the use of tracking and counting through a Kalman filters bank.

### 4.1. People Detection Using a MobileNet-SSD

This section explains how it works and why this network has been chosen for this system. The chosen network is a MobileNet-SSD, a MobileNet architecture [14], which uses the SSD [45] method for object detection. The architecture that forms this network is very similar to the one used in the VGG-16 [54]. The main difference is the replacement of the VGG-16 module by the MobileNet module. The reasons for using this architecture are two-fold: firstly because a fast architecture was needed, which could be implemented with selected algorithms such as SSD; and secondly, the resource consumption was low, because the devices used in this project are portable and its hardware is not as powerful as a high-performance PC. For this reason, MobileNet architecture was chosen, because its main feature is the speed of computing and the use of a type of convolutional layers that allow the use of fewer resources.

**Figure 3:**
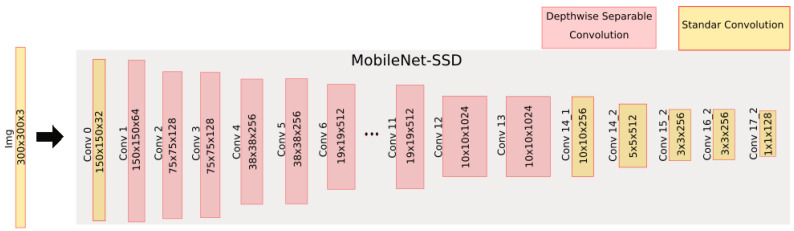
Data flow and multiple thread processing.

The architecture used is shown in Figure 3. It can be seen that at the beginning of the network there is the MobileNet module, composed of 35 convolutional layers, which are responsible for the feature extraction. A set of five layers of these 35 are in charge of carrying out the classification of objects applying an SSD.

This architecture reduces the intensity in data processing due to the use of separable depthwise and pointwise convolutional layers.

Thus, we compared the computational cost of a standard convolution used in any type of architecture such as the VGG-16 with respect to the operations required in the MobileNet convolutions.

**Figure 4:**
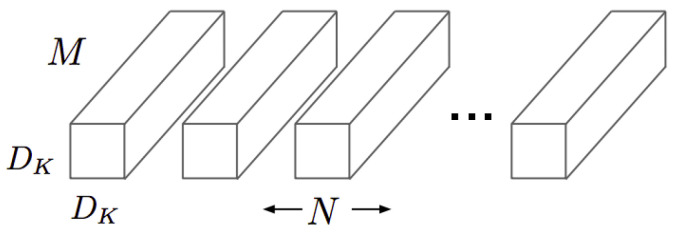
Standard convolution kernels [14].

To measure the computational cost of a standard convolution, first it is necessary to know the size of the kernels that compose it. Standard convolution kernels can be expressed graphically as shown in Figure 4, where the number of channels in the image is *M*, the number of kernels needed is *N*, which corresponds to the number of output channels, and the size of the kernels is $D_{k}\cdot D_{k}$.

For a $D_{F}\cdot D_{F}$ image, the computational cost of standard convolution $\left(CC_{ORD}\right)$ is defined in Equation (1):

(1) $$CC_{ORD}=D_{k}\cdot D_{k}\cdot M\cdot N\cdot D_{F}\cdot D_{F}$$

The following is the same way that the computational cost of the standard convolution was calculated. The study of the computational cost of the DW and pointwise was carried out.

**Figure 5:**
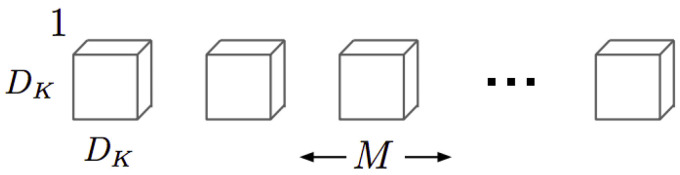
Depthwise convolution kernels [14].

The depthwise kernels have the structure shown in Figure 5. Where *M* is the number of the input channel image and DK is the size of the kernels. It must be noted that the depthwise output size is one, so at the network output there is only one kernel. This means that in the expression of the computational cost (2), *N* is equal to 1.

For an input image of $D_{F}\cdot D_{F}$ size, the computational cost of a depthwise convolution $\left(CC_{DW}\right)$ is defined in Equation (2):

(2) $$CC_{DW}=D_{K}^{2}\cdot M\cdot D_{F}^{2}$$

**Figure 6:**
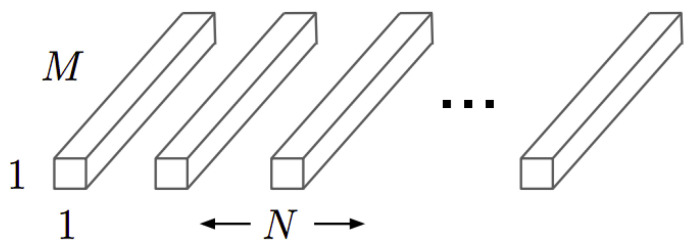
Pointwise convolution kernels [14].

The pointwise kernels have the structure as that shown in Figure 6. Where N is the number of kernels, M is the number of channels in the input image and the kernel size is 1 × 1. This is why this type of convolution is called a point convolution.

For an image of size $D_{F}\cdot D_{F}$, the computational cost of pointwise convolution $\left(CC_{DP}\right)$ is defined in Equation (3):

(3) $$CC_{DP}=MN\cdot D_{F}\cdot D_{F}$$

The combination of the computational cost of depthwise and pointwise is called depthwise separable with-convolution, which was first named in [14]. Thus, the separable depthwise with-convolution can be expressed as the sum of depthwise and pointwise (Equation (4)):

(4) $$CC_{DW+DP}=D_{K}\cdot D_{K}\cdot M\cdot D_{F}\cdot D_{F}+M\cdot N\cdot D_{F}\cdot D_{F}$$

In Equation (5), the computational cost of the separable depthwise convolution and the ordinary convolution are mathematically compared. As it can be observed, the computational cost of an ordinary convolution is greater than the one separable depthwise convolution. This allows the extraction of features to be faster and equally efficient:

(5) $$\frac{CC_{DW+DP}}{CC_{ORD}}=\frac{D_{K}^{2}\cdot M\cdot D_{F}^{2}+M\cdot N\cdot D_{F}^{2}}{D_{k}^{2}\cdot M\cdot N\cdot D_{F}^{2}}=\frac{1}{N}+\frac{1}{D_{K}^{2}}$$

As shown in the previous equation, this system is computationally lighter, fits perfectly on embedded hardware, facilitates portability and can be executed in real time. Furthermore, this architecture will run on the VPU, performing the inference in a more optimal way than on CPU.

#### Network Features

The network used for the detection algorithm has been implemented by means of the Keras API of Tensorflow [55]. To check that the network was correctly trained, it was compared with the existing caffe model. To train the network, three datasets were used. First, a scratch training was performed with MS-COCO [40] and then fine-tuning was performed with Pascal VOC 2007 [38] and 2012 [39] and evaluated with VOC 2007. The average accuracy compared to the caffe model confirms that the model has been correctly replicated in Keras. The Table 1 shows the mean precision with the caffe model and the Keras model.

For the training of the network, we used 500 epochs with a batch size of 32. An Adam optimizer with a learning rate of 1 × 10${}^{-3}$ and a decay of 5 × 10${}^{-4}$ was used.

Once the network is trained, the model weights will be processed by the model optimizer of the OpenVino framework as explained in the previous Section 3, extracting the IR files that will be used to form the network through the OpenVino engine.

### 4.2. People Tracking Using a Kalman Filter Bank

**Figure 7:**
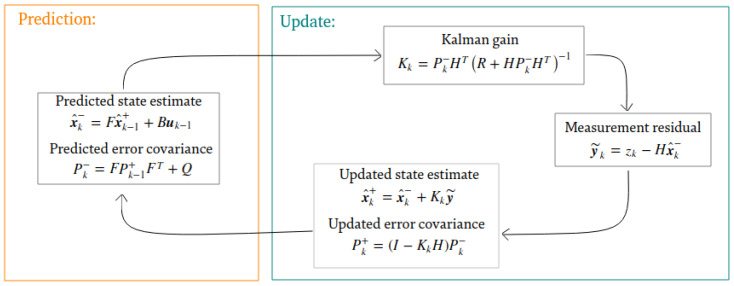
Kalman structure.

For the tracking of persons in our detection system, we resorted to the use of Kalman filters. The main reasons for the use of this type of filters are: the robustness to overlapping and the low computational cost of the filter. The principles and calculations of the Kalman filter are widely presented in the literature [56,57,58]. For our system, it is assumed that the movement of people will be with a constant and linear velocity. The state prediction and updated state equations are shown in Figure 7. These equations refer to the tracking of a single individual by a Kalman filter. Thus, for each detection that is performed on the MobileNet-SSD, a Kalman filter will be tracking each object.

The sequence of work performed for the Kalman filter bank is as follows:

- Tracker creation: when an object is first detected in the scene for N (this number is configurable) consecutive frames, the system must assign a Kalman filter to that new object;
- Object association: the system must be able to correctly identify the detections made by the detection system with the already assigned Kalman filters. This means that for each object detected, its Kalman filter must be associated;
- Kalman filter iteration: once the measurements of each object have been delivered to each Kalman filter, the iteration of each one of the filters must be done to fulfil the prediction and correction stages. If a previously identified object does not appear in the image due to an occlusion or a failure of the detector, the Kalman filter can use the estimated values until the object appears again in the scene;
- Filter elimination: in the case that a previously identified object has not been detected during a period of time, either because it has disappeared from the scene or because the detector is not able to identify it, the system must be able to finish the estimator associated to that object.

This tracking module will receive information directly from the MobileNet-SSD in the form of the bounding boxes of the detected objects. For each detected object with the MobileNet-SSD and exceeding a detection threshold, a Kalman filter will be assigned.

### 4.3. Pipeline Operation

**Figure 8:**
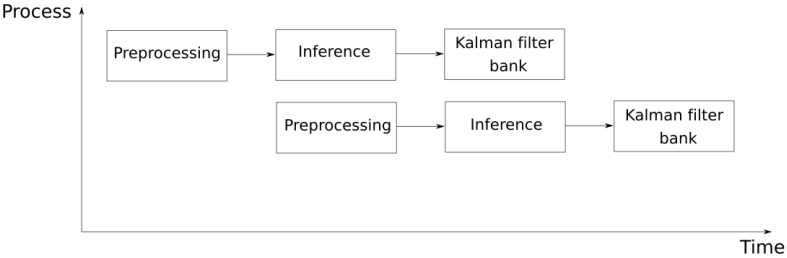
Pipelined stages.

The pipeline operation shown in Figure 2 was designed to parallelise each of the elements that form the system. The main elements that form the architecture are the preprocessing, the Kalman filter and the MobileNet-SSD inference. This parallelisation allows the processing of more than one video stream at the same time, since the inference is separated from the CPU processing. This inference is processed through the use of VPUs. These devices permit the processing of more than one inference at a time and also in a more optimal way than through CPUs. Figure 8 shows the structure of the processes that make the system up, a structure which can be parallelised as much as the hardware allows. This permits not only the processing of more video streams but also a more efficient use of the hardware systems that make the device up.

## 5. Results

The main objective of the contribution presented in the paper is to propose a portable and fast embedded system for detection, tracking and counting people.

**Figure 9:**
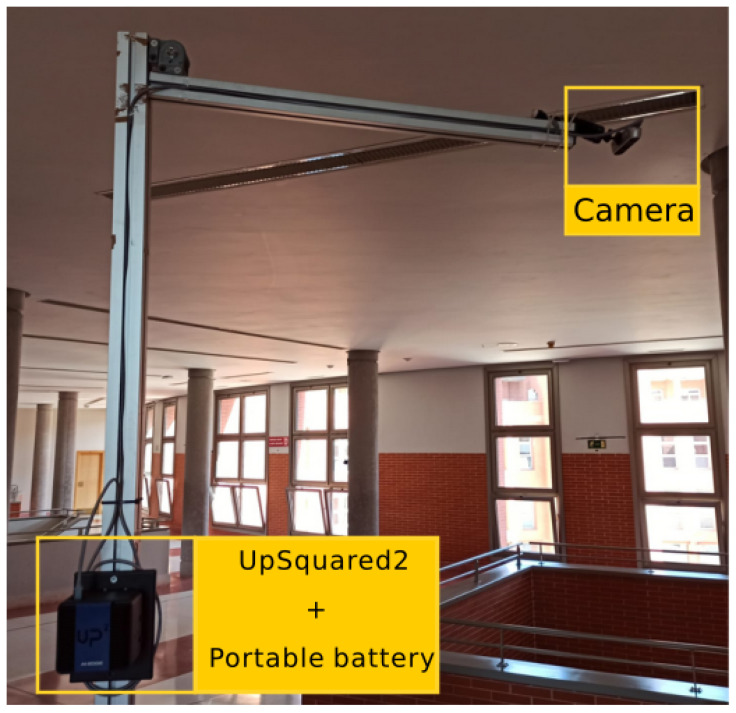
Mounted test system.

When talking about portability, we mean two goals: firstly, that it can be easily installed anywhere, as shown in Figure 9, and secondly, that it can be maintained and correctly operated without a connection to mains power, i.e., it can be powered by portable batteries.

The results section is divided into two parts. The first part describes the experimental setup, the datasets used and the edge hardware modules. The second part shows the comparison of obtained results with other similar research works. As mentioned below, the target features a portable system with robust detection and capable of running in real time. To evaluate these requirements, the following characteristics are analysed:

- Reliability and accuracy of the people detection of the proposed architecture compared with other state-of-the-art detection systems;
- The number of video streams that can be processed in real time using the edge hardware modules;
- The power consumption.

### 5.1. Experimental Setup

**Figure 10:**
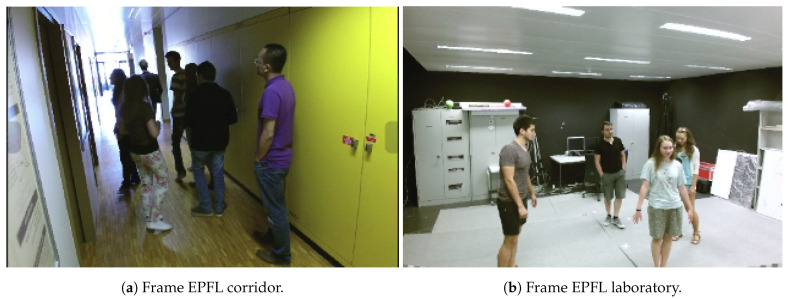
Different scenarios of the EPFL dataset.

For the system evaluation, the EPFL [59] dataset was used. The characteristics of this dataset fit a possible real scenario in which there are overlaps, large numbers of people and changes in the illumination. This dataset consists of two environments: the first one is a laboratory (Figure 10b), which is a large space in which there is a set of four people overlapping each other and changing their position in the image, moving away from and towards the camera. The second scenario is a university corridor (Figure 10a), in which there are up to eight people in a small space, at different distances from the camera, with changes in lighting between the different videos and with a high number of overlapping.

To evaluate the part concerning people detection, the precision and recall metrics were extracted, and through these values, we calculated the precision–recall(PR) curve. To check that the proposed system is as precise as those in the state of the art, we compared the PR curve with different methods, as shown in Table 2, and as explained below:

- ACF [60]: uses a RGB detector from [60], based on AdaBoost and aggregate channel features [61] to give a sense of what a state-of-the-art detector that does not use depth can do on these sequences;
- PCL-MUNARO [62]: uses a RGB-D detector from [62], based on modified HOG features on regions extracted by depth segmentation;
- DPOM [59]: is based on the detection of human presence on the ground plane using Bayesian inference. It is also based on the elimination of the ground plane from the image and the clustering of non-eliminated points as possible detections. When it has grouped all regions, the algorithm terminates;
- YOLO-v3 [63]: is a CNN that uses RGB information for object detection. The architecture is trained with the COCO network 2017 [40]. The parameters used to configure the architecture were an input image size of 416 × 416, nine anchors, and all the COCO;
- YOLO-depth: is a CNN based on [63] and re-trained to only work with depth information. The parameters used to configure the architecture were an input image size of 416 × 416, nine anchors, and only with the person class.

As explained in the previous Section 4.1, a MobileNet-SSD network trained with COCO datasets and fine tuned with VOC 2007 and VOC 2012 was used.

In addition to its performance, the system was also evaluated in terms of energy consumption on the UpSquared2 device. This edge device features an Intel Atom x7-E3950 microprocessor with 8 GB RAM memory and 64 GB embedded MultiMediaCard (eMMC) ROM. It also integrates a VPU MyriadX module onto the device’s motherboard. Using the OpenVino framework for the CNN inference, different measurements were obtained for the CPU and VPU implementations.

The OpenVino format model was configured to FP16, which was supported by VPU. Once the training was finished, we used part of the videos of the EPFL dataset to adjust the detection threshold of the system to the optimal value. The precision-recall curves were used to optimise the threshold value. The metrics extracted from EPFL were compared with other similar research works.

### 5.2. Precision Results in People Detection and Tracking

The performance of the software system for people detection was evaluated in this section. As previously mentioned, the system is composed of a MobileNet architecture with an SSD method. In addition, a Kalman filters bank was added to provide robustness and flexibility in the case of overlaps, allowing the tracking of the detected objects. As mentioned in the previous section, our system was compared with different person detection models. To this end, we used [64] paper to extract the precision–recall curve when they were processing EPFL dataset.

**Figure 11:**
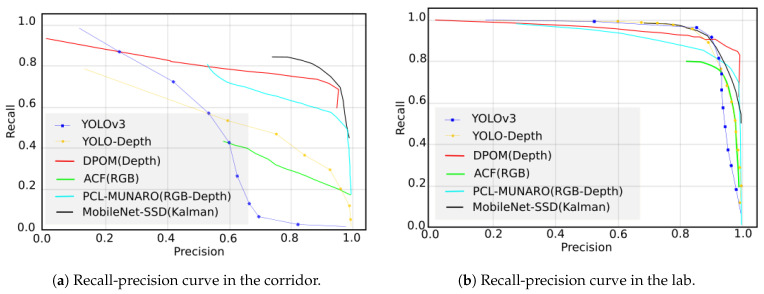
Recall-precision curves for different environments in EPFL dataset.

Figure 11 shows the different methods used in the two scenarios available in the EPFL dataset. Results were extracted with our system under the same conditions, and the same threshold-overlap was used in the different methods of the 40% dataset. The curve designed for our system is shown in black.

From Figure 11, it is observed that in the laboratory scenario (Figure 11b), all the systems have a similar performance. However, in the corridor scenario (Figure 11a), it was observed that the best results were those of the DPOM algorithm and the MobileNet-SSD with the Kalman filter.

The average precision and recall results of the two scenarios in EPFL are shown in the Table 3. The method used in this paper is MobileNet-SSD + Kalman, highlighted in bold. The highest scores for each evaluated metric are underlined.

As can be seen, the metric values exceed 80% in all cases, although there is a difference in the performance between the corridor and the laboratory. This is due to the fact that the number of overlaps and the number of people in the corridor videos are greater than in the laboratory, making detection more difficult.

### 5.3. AI Edge Computing Performance

In this section, we present the temporal results of the system processing in both CPU and VPU hardware modules. For this purpose, we used the frames with the largest number of people, in which the inference has a higher computational cost, placing the measurements in the most restrictive cases. In addition, measurements have been made on another larger network such as the YOLO-v3, in order to compare the computational cost with the MobileNet-SSD. The measurements concerning the inference of the CNNs were obtained from both the UpSquared2 CPU and on the VPU. These results are shown in Table 4.

Several conclusions can be extracted from Table 4: MobileNet-SSD is executed faster than Yolo-v3 in both types of HW. The acceleration ratio is almost the same in the CPU and VPU modules. The time execution of MobileNet-SSD is 29.3% and 28.9% of the total time execution of the YOLO-v3 inference for CPU and VPU, respectively.

The optimisation provided by the Myriad-X device with respect to inference, in both CNNs, represents about a 64% reduction in the computational cost. The use of VPUs in the AI inference at edge nodes provides a substantial improvement in time execution with a lower computational cost. Considering the accuracy results of MobileNet-SSD are similar to those of YOLO-v3 (see Table 3), the lower computational cost for the MobileNet-SSD makes it suitable for our system. In addition to the inference execution, the other processes, preprocessing and execution of Kalman filters bank, are always performed in the CPU with the following corresponding execution times: 0.48 ms and 5.03 ms, respectively.

After showing the execution times of the modules of the system independently, the use of the system with more than one video flow was theoretically analysed. For this study, we took into account the limitations of the hardware when executing processes simultaneously. For this reason, the system was first analysed using only the CPU of the UpSquared2, which uses an Atom x7-E3950 with four cores. This means that it can launch up to four processes at the same time. For the development of the system, the CPU while running has already occupied two out of its four cores through the use of the GUI of the operating system and the IDE used to run the system. That is why when analysing the processes executed on the CPU, only the two remaining cores are used.

**Figure 12:**
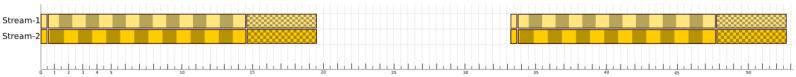
Stream processing schedule using CPU cores.

Figure 12 shows the execution schedule using the two cores. As shown, up to two video streams can be launched at 30 fps. As can be seen, the maximum that can be parallelised by CPU execution in 33.3 ms, are two video streams, and the CPU usage while receiving camera frames does not exceed 58.83%.

In the temporal analysis shown in Figure 12, the algorithm and processes are executed only using the CPU-cores. The graph shows the temporal occupation of the different processes. The first box is the preprocessing. The second box corresponds to the inference (box with a striped background). The third box represent the execution of the Kalman analysis (dotted background). The processes executed by the same device are shown in the same colour in the graph. As in this case, there are only two cores running the algorithm, the light yellow corresponds to core 1 and the dark yellow to core 2. As can be seen in Figure 12, the parallelisation is minimal, being able to process up to two video streams at 30 fps, i.e., one frame every 33 ms, with a latency value of 19 ms.

**Figure 13:**
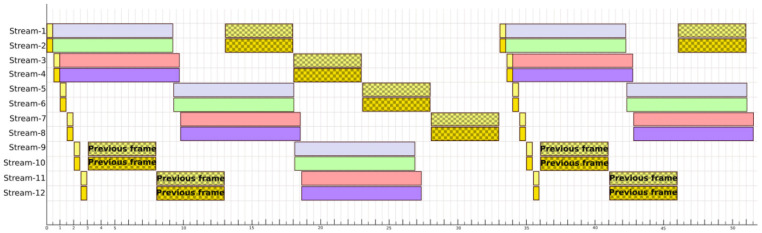
Stream processing schedule using CPU and VPU cores.

Now, the same problem is faced but this time using a VPU for the inference processing. The VPU used is the Myriad-X, which allows the simultaneous inference of up to four images in an optimal way. By rearranging how to run the different processes on the VPU cores available in the UpSquared2, 12 video streams can be analysed at 30 fps as shown in Figure 13. In the chronograph, the execution of the cores is represented with a light yellow and a dark yellow tone. When the preprocessing is executed it has a smooth background and when the Kalman filters bank is executed it has a background with dots. The four inferences made by the VPU are represented with a plain background with the colours grey, green, red and blue. Knowing this, it can be seen in the graph that the Kalman processing of the last four video streams must overlap with the beginning of the next frame. The cores corresponding to the CPU have a utilisation of 100% during the 33 ms between frames. On the other hand, the VPU has a utilisation of 73% between frames. A much tighter and more optimal parallelisation is observed compared when only using CPUs.

The following Algorithm 1 describes the pseudo-code of execution. The three main modules (CPU-core1, CPU-core2 and VPU-cores) are working in parallel, processing the different video streams. The CPU-core1 processes the odd video streams, while CPU-core2 replicates the same behaviour with even video streams. The execution in the UpSquared2 is divided in four threads. Two main threads are responsible for the management of the application, drivers, etc. The two other threads are for CPU-cores processing (CPU-core1 and CPU-core2) while the CNN inference is computed via the VPU-cores SHAVE. The communication between threads is done via global buffers taking into account whether or not the prior processing has finished.
**Algorithm 1** CPU and VPU processing.
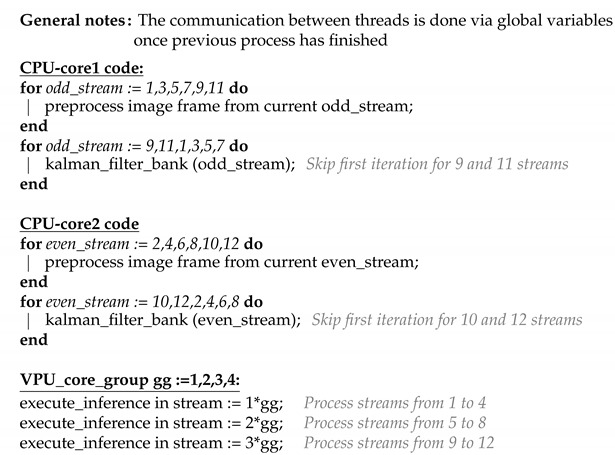


As it can be seen, with a proper management of code and tread execution the edge node with a VPU can process up to four times more information than just using the system CPU. The following section discusses the energy consumption of the smart edge node as it can be powered with a portable power supply system.

### 5.4. Power Consumption

In this section, a study of the embedded system power consumption is carried out. For this purpose, the system has been powered by an external power supply to the device, in order to monitor the power consumption. The UpSquared2 is a device that is powered with a 5 V input voltage, where the variations of current sunk in different moments have been studied. The consumption generated by the VPU has also been evaluated separately.

Therefore, the consumption of the node has been measured in different situations: idle, executing the algorithm only in the CPU and finally using the CPU plus the VPU. The power consumption values obtained are shown in Table 5.

As shown in Table 5, the power consumption using the VPU (“AI on VPU + TRACK on CPU”) is higher than using the CPU alone (“AI + TRACK on CPU”). The difference can be seen in the power consumed. Nevertheless, it was also observed that the global energy drained with the CPU+VPU operation (“AI on VPU + TRACK on CPU”) was 25% less than the one drained when using only the CPU (“AI + TRACK on CPU”).

Considering the number of video streams that might be processed using the VPU (up to 12 cameras), the consumption per videofeed would be 17.08 W*ms, a very reduced figure compared with the 117.13 W*ms obtained when only using the CPU processing two video streams.

Previously obtained values were compared with other similar works, such as that of [65], whose architecture uses a Nano Jetson as an embedded system executing a YOLOv3-tiny network. This network is similar to MobileNet-SSD in size and computational cost. Boschi shows that its system consumes 9 W, providing a performance of up to 9 fps, whereas the proposed system in this paper using the novel edge technology (VPUs) consumes 14.41 W and allows the processing of more than 30 fps. The power consumption per fps relationship is lower in our system than in other proposals, achieving a real-time execution with a moderate power consumption, and being able to be supplied with portable batteries.

## 6. Conclusions

The paper proposes a portable video surveillance system with AI CNN processing at the edge, which can detect and track people in a robust and reliable way. The novel computer technology used at the edge is VPU hardware modules, which allow performing the CNN inference faster and more efficiently than a CPU in low-end devices. The designed system permits implementing a computer vision application with low power consumption and high computational performance. To achieve this, an embedded device UpSquared2 was used, which integrates a Myriad-X VPU where the chosen CNN, a MobileNet-SSD, is executed. One of the requirements was the real-time execution of the system. To achieve real-time, we used the OpenVino 2020.2 framework, which allows an optimisation of the CNN, streamlining its inference and facilitating the execution on the VPU. In addition, this framework allows the parallel inference of the CNN on the VPU SHAVES. The inference was executed both on VPU and CPU. An improvement in the processing speed of 64.59% was obtained when the processing was done with the VPU instead the CPU.

The paper also presents a software system based on deep learning (MobileNet-SSD) and Kalman filter banks. The system was compared with other state-of-the-art machine learning (ACF, PCL-MUNARO) and deep learning (DPOM, YOLOv3, YOLO-depth) methods for people detection. The achieved results are in line with the current state of the art, having a precision of 81.43% and a recall of 80.6% in the EPFL-corridor dataset, while in the EPFL-laboratory, both precision and recall were above 87%.

Another important point of the system is the processing of multiple video streams in real time (+30 fps), supporting up to 12 streams with the hardware provided by the UpSquared2 using the integrated VPU. In addition to this, the system has been designed to be portable and easy to manipulate, both in the adjustment of internal software parameters such as adjusting the region of interest and the detection threshold, as well as the power consumption of the system. Having an average power consumption of 12 W, with peaks of up to 15 W, when running AI inference on the VPU. Being able to power the system with portable batteries or renewable systems provides great flexibility in distributed surveillance camera systems.

## Figures and Tables

**Table 1:** Mean average precision comparison.

Mean Average Precision (mAP)		
Network Used	Original Caffe Model	Keras Model
MobileNet-SSD300 “COCO+VOC07+VOC12”	72.5	72.7

**Table 2:** Evaluated state-of-the-art methods.

Solutions	Methods	Input Information
Classic	ACF [60]	RGB
	PCL-MUNARO [62]	RGB
DNN	DPOM [59]	Depth
	YOLO-V3 [63]	RGB
	YOLO-depth [63]	Depth

**Table 3:** Recall and precision of EPFL dataset.

Methods	EPFL-LAB		EPFL-CORRIDOR	
	Precision	Recall	Precision	Recall
**MobileNet-SSD + Kalman**	**87.82**	**88.14**	**81.3**	**80.6**
ACF	83.8	86.4	66.3	40.3
DPOM	98.5	85.4	96.3	70.9
PCL-MUNARO	88.61	82.36	92	56
YOLOv3	89.7	90.3	58.6	59.8
YOLO-depth	89.8	88.6	78.4	47.9

**Table 4:** Inference computational cost (in ms) for the different algorithms using CPU or VPU.

Inference Performance on UpSquared2	t_CPU_ (ms)	t_VPU_ (ms)
MobileNet-SSD	13.93	8.71
YOLO-v3	47.54	30.07

**Table 5:** Power and energy consumption values depending on the execution mode CPU/VPU.

Device/Mode	I Mean [A]	Pot [W]	Time [ms]	Energy [W·ms]
IDLE	1.08	5.41	-	-
AI + TRACK on CPU	2.41	12.05	19.44	234.25
AI on VPU + TRACK on CPU	2.88	14.41	14.22	204.91

## Data Availability

Publicly available datasets were analyzed in this study. This data can be found here: [https://www.epfl.ch/labs/cvlab/data/data-rgbd-pedestrian/ (accessed on 11 March 2021)].

## Abbreviations

**Abbreviation**	**Meaning**
AI	Artificial intelligence
VPU	Vision processor unit
IoT	Internet of things
DNN	Deep neural network
GPU	Graphical processing unit
SSD	Single shot detection
HOG	Histogram of gradient
SVM	Support vector machine
PCA	Principal components analysis
CNN	Convolutional neural network
R-CNN	Recursive-convolutional neural network
YOLO	You only look once
NCS	Neural computer stick
mAP	Mean average precision
